# Control Strategy on Hypertension in Chinese Medicine

**DOI:** 10.1155/2012/284847

**Published:** 2011-12-12

**Authors:** Jie Wang, Xingjiang Xiong

**Affiliations:** Department of Cardiology, Guang'anmen Hospital, China Academy of Chinese Medical Sciences, Beijing 100053, China

## Abstract

Hypertension is a clinical common disease, with high mortality and disability. Although there have also been significant advances in therapeutic concepts and measures, it has shown a certain value and significance in the treatment of Chinese medicine. The control strategy on hypertension is described from the following aspects such as differentiation of symptoms, pathogenesis, formula syndrome, and herb syndrome. As the common clinical manifestations of hypertension are dizziness, headache, fatigue, lassitude in the loins and knees, and so on, the pathogeneses of them are analysed. The author found that the main pathogenesis of the disease is heat, excessive fluid, and deficiency, which occurred incorporatively and interacted with each other in patients. Although the pathogenesis of the disease is complicated, the distribution of formula syndromes and herb syndromes is regular. The common formula syndromes include *Banxia Baishu Tianma Tang* (Decoction of *Pinellia ternata, Atractylodes* and *Gastrodia elata*), *Da Chaihu Tang* (Major Bupleurum Decoction), and *Liu Wei Dihuang Wan* (Pill of *Rehmannia*). And the common herb syndromes include Tian Ma (*Gastrodia elata*) syndrome, Sheng Di Huang (Radix Rehmanniae) syndrome, Niu Xi (*Achyranthes Root*) syndrome, and Chuan Xiong (*Ligusticum wallichii*) syndrome.

## 1. Introduction

Hypertension is a clinical common disease, with high mortality and disability. Additionally, it is also an independent risk factor for stroke, coronary heart disease, heart failure, renal insufficiency, peripheral vascular diseases, early death, and many other major diseases [1]. In numerous studies, it is shown that around 30% of the population died from cardiovascular and cerebrovascular events, in which 62% of acute stroke events and 49% of cardiovascular events were directly caused by hypertension. There are about 1 billion hypertensive patients in the world. It was demonstrated that there were about 200 million hypertensive patients in China with more than 10 million patients increased annually [2, 3] in Cardiovascular Report 2006 in China. The antihypertensive treatment has made great progress in modern medicine. The therapeutic drugs include six classes of antihypertensive agents and fixed compound preparation. There have also been significant advances in therapeutic concepts and measures, including paying more attention to the control of earlier-stage hypertension and elevated systolic blood pressure, optimization of combination therapeutic scheme, and new strategy of antihypertensive treatment combined with lipid-lowering therapy [4]. Despite the availability of six classes of antihypertensive agents, control of blood pressure and improving patients' quality of life remain unsatisfactory, and “three lows” status quo, low awareness, low treatment, and low control, still exist. As the complexity of hypertension, there are still some issues to be resolved. For instance, most patients are prescribed single or combined antihypertensive drugs for long-term even lifetime. It also has been confirmed that the emergence of adverse effect and irrational drug use increase the difficulty and dissatisfaction in treatment [5]. Currently, it has shown a certain value and significance in the treatment of Chinese medicine. Now the understandings of hypertension in Chinese medicine and control strategies with classical prescriptions are described as follow.

## 2. Differentiation of Symptoms

Symptoms are not only the basis for syndrome differential diagnosis but also the therapeutic targets. As Chinese medicine had no idea of blood pressure originally, it treated patients mainly by differentiating the syndromes according to the symptoms and signs caused by hypertension and paid more attention to relieve symptoms and improve the quality of life. The most common clinical manifestations of hypertension are dizziness, headache, fatigue, shortness of breath, lassitude in the loins and knees, memory loss, dry eye, palpitation, and so on [6]. 

The disease belongs to “vertigo” in Chinese medicine. The main pathogenesis of vertigo is stagnation of phlegm, excess of liver yang, deficiency of qi and blood, liver-kidney yin deficiency, and stagnation of blood. In addition, there are important pathogenesis such as excessive fluid and pathogenic factor in Shaoyang meridians according to the clinical experience in* Shang Han Lun *(Treatise on Febrile Diseases). Dizziness caused by excessive fluid syndrome is always accompanied by chest distress, palpitation, nausea, and vomiting. Paroxysmal dizziness is one of the typical symptoms in Shaoyang syndrome. Persistent dizziness, headache, nausea, and vomiting can be found in Shaoyang syndrome accompanied by heat and excessive fluid syndrome. 

The syndrome differentiation of headache is similar to vertigo. The main pathogenesis of headache is excess of liver yang, deficiency of qi and blood, kidney deficiency, stagnation of phlegm and blood. We found that headache is caused by the flaring-up of fire in patients with hypertension. Flaring-up of stomach fire and intestinal fire followed by meridians are also very common, besides excess of liver yang. Severe headache generally suggests blood stasis; therefore, blood-activating and stasis-dissolving drugs should be used to relieve pain quickly. Moreover, cold accumulation in the liver and stomach should not be neglected, which can result in severe headache in the disease. 

Weakness and fatigue are extremely common symptoms of the disease. Generally speaking, it is caused by deficiency of qi, blood, yin, and yang. However, they are not the characteristic symptoms of deficiency syndrome. Some are caused by dampness and water accumulated inside. As the characteristic of pathogenic dampness is weight and sticky, which leads to stagnation of the functional qi and consumption of yang qi, weakness, fatigue, heaviness in head and body, and lassitude in loins and limbs often appear. 

In addition, although lassitude in loins and limbs is the characteristic symptom of kidney deficiency, excessive fluid is also an important factor of the symptom. *Fu Ling* (*Poria cocos*) and *Bai Shu * (*Atractylodes*) contained in *Gancao Ganjiang Fuling Baishu Tang* (Decoction of *Glycyrrhiza*, Dried Ginger, *Poria cocos*, and *Atractylodes*) treating lassitude in loins and limbs can invigorate spleen to resolve dampness. *Fu Ling* (*Poria cocos*) and *Ze Xie* (*Alisma*) contained in *Liu Wei Dihuang Wan *(Pill of *Rehmannia*) and *Shen Qi Wan* (Pill of Kidney Qi) have the identical effection.

## 3. Differentiation of Pathogenesis

The identification of the etiology and pathogenesis of hypertension in TCM viewpoint is directly related to the syndrome differentiation and therapeutic methods. According to the traditional view, the pathogenesis of hypertension is considered as founding on yin deficiency, with yang hyperactivity in the superficiality and phlegm-dampness and blood stasis penetrating all along; therefore, the basic therapeutic methods should be to supplement qi and nourish yin [4]. However, due to the widely used antihypertensive agents, the progression of the disease was blocked in time, which leads to great changing of the natural history and pathogenesis of hypertension. Therefore, we should not only pay attention to yin deficiency and yang hyperactivity syndrome but also follow the pathogenesis and syndromes of the illness with interest in the recognition of the pathogenesis of hypertension and treat the disease according to clinical symptoms. The author found that the main pathogenesis of the disease is heat, excessive fluid, and deficiency, which occurred incorporatively and interacted with each other in patients. 

Heat syndrome can be found in various stages of hypertension, especially when target organ damage is not found. Heat syndrome includes liver fire, heart fire, stomach fire, and intestinal fire. Liver fire syndrome often shows clinical symptoms such as vertigo, headache, facial flush with perspires, conjunctival congestion, bitter taste in the mouth, irritability, wiry-rapid-powerful pulse, or powerful cunkou pulse alone, or wiry and long pulse even well beyond cunkou pulse. *Longdan Xie Gan Tang* (Decoction of Radix Gentianae for Purging Liver Fire) noted in *Yi Fang Ji Jie* (Collection of Prescriptions with Notes), *Tianma Gouteng Yin * (Decoction of *Gastrodia* and *Uncaria*) noted in *Za Bing Zheng Zhi Xin Yi *(New Meanings in Syndrome and Therapy of Miscellaneous Diseases), and *San Cao Jiang Ya Tang* (Decoction of Antihypertensive Effect) which is Professor Liu Duzhou's experienced prescription containing Long Dan Cao (Gentian), Xia Ku Cao (*Prunella vulgaris*), Yi Mu Cao (*Leonurus japonicus*), Shao Yao (Chinese Peony), and Gan Cao (Glycyrrhiza) all can be used for clearing liver fire and lowering blood pressure. Patients with heart fire syndrome often present with distraction, distress in chest, vexation, palpitation, nervousness, insomnia and dreaminess, hard to sleep, and easy to wake. *Zhizi Chi Tang *(Decoction of *Gardenia* and Lobster Sauce) and *Huanglian Jie Du Tang* (Detoxicant Decoction of *Coptis*) all can be used for clearing away heart fire and lowering blood pressure. Patients with stomach fire syndrome often present with dry mouth, thirst with desire for cold drinks, easy to starve, foul breath, smelly stool, and right guan pulse powerful alone. *Bai Hu Tang* (White Tiger Decoction) can be used to clear away stomach fire and lower blood pressure. Intestinal fire syndrome often shows foul breath, constipation, abdominal distension and pain, strength, and deep-hidden-powerful pulse. *Da Chaihu Tang* (Major *Bupleurum* Decoction) can be used for clearing away intestinal fire, dredging intestines, and descending turbid substance. Liver fire and heart fire often appear simultaneously, which result in hyperactivity of heart fire and liver fire syndrome in the disease. In addition, the liver restricting the spleen and stomach may also result in hyperactivity of liver fire, stomach fire, and intestinal fire. 

Fluid retention syndrome is a special type of the disease, containing morbid fluid retained in Shangjiao, Zhongjiao, and Xiajiao syndromes, cold fluid retention syndrome, fluid retention turning into heat syndrome, and downward flow of damp-heat syndrome. Morbid fluid retained in Shangjiao syndrome often shows dizziness aggravated by body position change, chest distress, palpitation, and cough. Morbid fluid retained in Zhongjiao syndrome often shows gastric distension, abdominal distension, nausea, vomiting, poor appetite, thirst without desire to drink, or not thirsty, daytime sleepiness, and greasy fur. Morbid fluid retained in Xiajiao syndrome often shows soreness, lumbar heaviness, low back pain, weakness and heaviness in lower extremity, edema, abnormal leucorrhea, dysuria and thick-greasy fur of tongue root. Cold fluid retention syndrome can appear retching, headache, cold hands and feet in syncope and dysphoria. Fluid retention turning into heat syndrome and downward flow of damp-heat syndrome can shows weakness and heaviness of lower extremity, thirst, yellow and ropiness leukorrhea, beriberi, and thick-greasy fur of tongue root. *Zexie Tang *(Decoction of American water Plantain),* Fuling Xingren Gancao Tang* (Decoction of *Poria cocos*, Almond, and *Glycyrrhiza*), *Wu Ling San* (Wuling Powder), *Ling Gui Zhu Gan Tang* (Decoction of Poria cocos, Cassia Twig, *Atractylodes macrocephala* and *Glycyrrhiza*), *Banxia Baishu Tianma Tang *(Decoction of *Pinellia ternata*, Atractylodes Macrocephala and *Gastrodia elata*), *Wu Zhu Yu Tang* (Decoction of *Evodia rutaecarpa*), *Er Miao Wan* (Ermiao Pill), *San Miao Wan* (Sanmiao Pill), and *Si Miao Wan* (Simiao Pill) all can be used for dissipating excessive fluid. 

Deficiency syndrome includes spleen deficiency syndrome and kidney deficiency syndrome. As spleen promotes transportation and transformation of dampness, excessive fluid retention is related to dysfunction of spleen. Cang Zhu (Rhizoma Atractylodes), Bai Zhu (*Atractylodes macrocephala*), Fu Ling (*Poria cocos*), Ze Xie (American water Plantain), and Gan Cao (*Glycyrrhiza*) all can be used for removing dampness by strengthening spleen and replenishing qi. Kidney deficiency syndrome includes kidney yin deficiency syndrome and kidney yang deficiency syndrome. As prolonged disease involves kidney, we found that kidney deficiency syndrome is always related to hypertension. The longer the course of illness, the higher the incidence rate of kidney deficiency syndrome. Furthermore, it is not to be neglected that the antihypertensive drugs can also lead to kidney deficiency. Diuretic and *β*-blocker have greater impact on sexual function than other antihypertensive drugs. Sexual function will decrease when the above two antihypertensive drugs are sustained using more than ten years. *Liu Wei Dihuang Wan *(Pill of *Rehmannia*), *Shen Qi Wan* (Pill of Kidney Qi), *Qi Ju Dihuang Wan* (Pill of Chinese Wolfberry, *Chrysanthemum*, and *Rehmannia*) and *Ji Sheng Shen Qi Wan* (Pill for Reinforcing Kidney Qi) all can be used for reinforcing kidney.

## 4. Differentiation of Formula Syndrome

Although the pathogenesis of the disease is complicated, the distribution of formula syndromes is regular. The common formula syndromes include *Banxia Baishu Tianma Tang *(Decoction of *Pinellia ternata*, *Atractylodes* and *Gastrodia elata*), *Da Chaihu Tang* (Major *Bupleurum* Decoction), and *Liu Wei Dihuang Wan *(Pill of *Rehmannia*). *Banxia Baishu Tianma Tang *(Decoction of *Pinellia ternata*, *Atractylodes macrocephala*, and *Gastrodia elata*) is the classical representative famous prescription for calming liver, suppressing liver yang hyperactivity, dissipating excessive fluid, and expelling phlegm in Chinese medicine. It has been used widely in clinical practice for treatment of hypertension, which is also the author's experienced prescription. Prestigious Chinese physicians such as Yue Meizhong and Jiang Erxun are skilled in using the decoction in both China and the world. Japan's Chinese physicians often use it to treat hypertensive patients with weakness of gastrointestinal function. It can treat liver fire and morbid fluid retained in Zhongjiao, the indications of which are dizziness, headache, palpitation, abundant sputum, or without phlegm, poor appetite, nausea, and vomiting aggravated by tooth brushing, abdominal distension, soreness, lumbar heaviness, heaviness in lower extremity, edema, dysuria, constipation, or loose stool, big and light tongue with greasy fur and slippery pulse. The decoction is suitable for patients lacking physical activity for a long time, especially for old women, the physical characteristics of which are obesity of abdominal type with yellow and white skin, soft muscle, ease of dizziness, and headache. It is often combined with *Zexie Tang *(Decoction of American water Plantain),* Wu Ling San* (Wuling Powder), and *Er Miao Wan* (Ermiao Pill) in treating hypertension. 

*Da Chaihu Tang* (Major *Bupleurum* Decoction) syndrome is a special type of the disease, which can be used for reconciling Shaoyang meridian and descending turbid substance. It has been used for treating liver fire, stomach fire, and intestinal fire. The composing herbs such as Chai Hu (*Bupleurum*) and Huang Qin (*Scutellaria*) can sooth liver and clear liver fire. Da Huang (Rhubarb), Zhi Shi (*Citrus aurantium*), and Shao Yao (Peony) can clear stomach fire and relieve constipation. Professor Huang Huang, coming from Nanjing University of Chinese Medicine, is skilled in using the decoction, the indications of which are dizziness, headache, facial flushing, conjunctival congestion, dry mouth, bitter taste in the mouth, halitosis, abdominal distension, constipation, yellow urine, red tongue, and wiry-rapid-powerful pulse. Professor Huang Huang pointed out that the physical characteristics are obesity type, strength, hard fullness, and distending pain of upper abdomin mostly accompanied by biliary-pancreatic diseases, poor appetite, nausea, vomiting, constipation, suppressive emotions, stress, sleep disorders, and so on. We found that the decoction is suitable for the young and middle-aged men with hypertension. Professor Huang Huang often combined *San Huang Xie Xin Tang* (Decoction of Sanhuang for Purging Heart Fire) to clear heart and liver fire [7]. 

*Liu Wei Dihuang Wan *(Pill of *Rehmannia*) is the classical representative famous prescription for nourishing kidney yin, which has been widely used for treating kidney deficiency and excessive fluid syndrome. The indications of the decoction include dizziness, headache, tinnitus, low back pain, lassitude in loins and legs, edema, big and light tongue with less fur, and deep thready pulse. Due to the speciality of the disease, two therapeutic principles should be followed. The first one is that herbs should be prescribed according to the rule of syndrome differentiation and treatment, and the second one is that the pharmacological action of herbs should act on the pathological mechanism of the disease. *Liu Wei Dihuang Wan *(Pill of *Rehmannia*) not only accords with the pathogenesis but also aims at the pathological mechanism. This compound prescription has broad pharmacological activities, especially the action of protecting vascular endothelial cells, dilating peripheral blood vessels, decreasing peripheral vascular resistance, lowing blood pressure synergistically, and delaying the development and progression of arteriosclerosis. If accompanied by blurred vision, Gou Qi (Chinese Wolfberry) and Ju Hua (*Chrysanthemum*) should be added into the prescription, which composed *Qi Ju Dihuang Wan* (Pill of Chinese Wolfberry, *Chrysanthemum,* and *Rehmannia*). If accompanied by facial flushing and conjunctival congestion, Tian Ma (*Gastrodia elata*) and Gou Teng (*Uncaria*) should be added into the prescription for calming liver and suppressing liver-yang hyperactivity.

## 5. Differentiation of Herb Syndrome

As the distribution of herb syndromes of hypertension is regular, we could find rules of formula syndromes from herb syndromes if we grasp the treatment rules of herb syndromes neatly and masterly. We found that the common herb syndromes include Tian Ma (*Gastrodia elata*) syndrome, Sheng Di Huang (Radix Rehmanniae) syndrome, Niu Xi (*Achyranthes root*) syndrome, and Chuan Xiong (*Ligusticum wallichii*) syndrome. 

Tian Ma (*Gastrodia elata*) can calm liver and suppress liver yang hyperactivity. The indications of Tian Ma (*Gastrodia elata*) are dizziness, tinnitus, distending feeling in head, headache, facial flushing and conjunctival congestion, the pathogenesis of which belongs to flaming up of liver fire and hyperactivity of liver yang. It is noteworthy that if dizziness and headache are more severe, the dose of Tian Ma (*Gastrodia elata*) must be larger to control blood pressure, and we often use 30 g, while routine dose such as 10 to 20 g has unsatisfactory results. The dose of Tian Ma (*Gastrodia elata*) in academician Chen Keji's experienced prescription “*Qing Xuan Jiang Ya Tang* (Decoction for Eliminating Vertigo and Lowering Blood Pressure)” is 30 g, while the dose of another key herb Gou Teng (*Uncaria*) is 30 to 60 g [8]. 

Sheng Di Huang (Radix Rehmanniae) could remove pathogenic heat from blood, nourish yin, and generate body fluid. In accordance with record of *Shengnong Ben Cao Jing* (Shennong's Classic of Meteria Medica), it can activate blood circulation. Yoshimasu T's “*Yao Zheng* (Indications of Herbs)” recorded that it can treat blood syndrome and fluid retention. Therefore, the indications of Sheng Di Huang (Radix Rehmanniae) are low back pain, lassitude in loins and legs, edema, hypodynamia, dry mouth, constipation, thin and less fur. We often use it to treat liver fire, intestinal fire, kidney fire, kidney deficiency, and excessive fluid. The dose of Sheng Di Huang (Radix Rehmanniae) needs our attention as well as Tian Ma (*Gastrodia elata*). Low dosees enrich yin and nourishes kidney, while large dose could activate blood to dredge vessels. According to the author's experience, the clinical dose is 60 to 120 g. It is not necessary to be too tense if patients present with gastrointestinal symptoms such as diarrhea and smelly stool after taking medicine, which means pathogenic fire is downgoing and eliminating. 

According to recordation in *Shengnong Ben Cao Jing* (Shennong's Classic of Meteria Medica), Niu Xi (*Achyranthes root*) can nourish liver and kidney, promote blood circulation to remove blood stasis, guide fire, and fluid downgoing. Therefore, dizziness, headache, facial flushing, conjunctival congestion, lassitude in loins and legs, and edema due to upward attack by qi and fire are the typical indications of Niu Xi (*Achyranthes root*). Professor Xu Wenhua, a prestigious Chinese physician of Jiangsu, is skilled in using Niu Xi (Achyranthes Root) treating pheochromocytoma, malignant abdominal tumors, and so on in clinical practice. The dose of Niu Xi (Achyranthes Root) is very large even up to 250 g sometimes. He had taken herbal decoction with Niu Xi (Achyranthes Root) of 200 g, and no serious side effect was found during the course of the treatment. Motivated by this, Professor Huang Huang often use large dose of Niu Xi (Achyranthes Root) to treat lower limb dropsy due to hemocirculatory disorder, cirrhosis ascites, hypertension patients with obesity, and so on. We often use it to treat liver fire, kidney deficiency, and fluid retention disease. Significant curative effect can occur when the dose is above 60 g, and simultaneous use of both Chuan Niu Xi (*Achyranthes bidentata*) and Huai Niu Xi (*Achyranthes bidentata Blume*) could improve the curative effect. 

Chuan Xiong (*Ligusticum wallichii*) could promote qi and activate blood circulation to relieve pain. According to recordation in* Shengnong Ben Cao Jing* (Shennong's Classic of Meteria Medica), it can treat headache. Famous Chinese physician Li Dongyuan also said that Chuan Xiong (*Ligusticum wallichii*) must be used in treating headache. Therefore, the classical indication of Chuan Xiong (*Ligusticum wallichii*) is headache. Severe headache always suggests qi stagnation, blood stasis, and meridians and collaterals impassable. The dose of Chuan Xiong (*Ligusticum wallichii*) also deserves attention greatly. We had treated one case of intractable migraine patients with Xue Fu Zhu Yu Tang (Decoction for Removing Blood Stasis), in which the dose of Chuan Xiong (*Ligusticum wallichii*) is 10 g. However, after taking 10 doses, the pain was not alleviated satisfactorily. It is found that routine dose such as 10 to 20 g cannot get satisfactory effect when headache is severe through many experiments afterwards. However, headache were relieved rapidly when the dosages was increased up to 30 g. 

## 6. Summary

Chinese medicine has been used for over 2500 years and has historically established itself as a system of a holistic medical care in China [9]. What is more, Chinese medicine and integrative medicine health provision in conventional medical clinic and hospital settings has emerged worldwide [10–13]. Seeing the body as a whole, not as separated systems, regarding human beings as more than just their physical bodies, practicing with emphasis on prevention, holoregulation, and comprehensive intervention, are all very strong principles of Chinese medicine, which works better and costs less than conventional medicine for the management of common diseases [14]. 

Blood pressure regulation involves multiple system interaction, such as kidneys, central nervous system (CNS), peripheral nervous system (PNS), and endothelial system. Hypertension is caused by a variety of factors such as the interaction of environment and heredity factors leading to disturbances of blood pressure regulation, the pathological mechanism of which includes sympathetic nervous system (SNS), renin-angiotensin-aldosterone system (RAAS), vasopressin (VP), nitric oxide (NO), endothelin (ET), adrenal medulla, and a variety of vasoactive peptide secreted by other endothelial cells and smooth muscle cells [15–17]. Although hypertension is a common cardiovascular disease, it is always combined with hyperlipidemia, coronary heart disease, diabetes, metabolic syndrome, and other diseases, which involve the cardiovascular, endocrine, neurologic, renal medicine, and other departments. Therefore, hypertension is a chronic and complex disease relating to systemic multiple systems. Besides lowering blood pressure steadily, the final purpose of Chinese medicine in treating hypertension is to reduce blood pressure variability and risk factors, protect hypertension target organs from the impairment, modulate factors difficult to control blood pressure, reduce the dosage of western medicine and abate the adverse reactions, relieve symptoms to improve patients' quality of life, improve long-term survival, and reduce the morbidity and mortality maximally [18]. It may be just what the scholars of Chinese medicine need to focus on, reflecting the advantages of Chinese medicine treatment. 

Although antihypertensive drugs have great advantage in reducing blood pressure rapidly with reliable effect, Chinese medicine can work at different levels and targets quite different from western drug which works relatively at few targets [19]. Researches had shown that Chinese herbs can regulate the function of RAAS, sympathetic-vagus nerve, and immune system, inhibit the level of inflammatory factor, prevent and reverse left ventricular hypertrophy caused by high blood pressure, influence vasoactive substances significantly, and so on [20–23]. Professor Li had observed the effect of Chinese medicine, Jiangya capsules, which was made up of Radix Cyathulae, Radix Achyranthis Bidentatae, *Pheretima, Laminaria japonica,* Rhizoma Gastrodiae, Rhizoma Chuanxiong, and so on, with multicenter, randomized, double-blind, positive controlled clinical design in elderly patients with isolated systolic hypertension (EISH). It showed that Chinese medical regimen had affirmative effect in treating EISH patients and could lower the systolic blood pressurea and improve quality of life and early renal impairment of the patients [24]. Professor Zhou found out that combined use of Xuezhikang or pravastatin with the antihypertensive therapy could increase the circulating endothelial progenitor cells number and improve their function in essential hypertensive patients with the blood pressure controlled by antihypertensive drugs, leading to benefits independent of pressure-lowering effects [25]. 

Furthermore, we found that there are laws for treatment according to many of years experience of large number of patients with hypertension. It has important significance in carefully analyzing common symptoms of hypertension, the etiology and pathogenesis, formula syndrome rules, herb syndrome rules, dose rules, combined diseases, and uncontrollable factors of blood pressure under the guidance of theory of Chinese medicine [26, 27].
